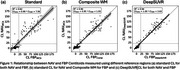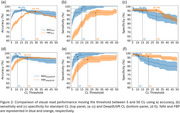# The impact of AI correction on Centiloid in a same subject visual read comparison of ^18^F‐Florbetapir and ^18^F‐NAV4694 Aβ PET

**DOI:** 10.1002/alz70856_106283

**Published:** 2026-01-07

**Authors:** Christopher C. Rowe, Ishara Paranawithana, Vincent Dore, Aurora Poon, H. B. Toh, Tanyaluck Thientunyakit, Tawika Kaewchur, Antony Sutherland, Kun Huang, Azadeh Feizpour, Jurgen Fripp, Victor L. Villemagne, Pierrick Bourgeat

**Affiliations:** ^1^ Department of Molecular Imaging & Therapy, Austin Health, Melbourne, VIC, Australia; ^2^ Florey Institute of Neuroscience and Mental Health, University of Melbourne, Melbourne, VIC, Australia; ^3^ CSIRO Health and Biosecurity, Australian E‐Health Research Centre, Parkville, VIC, Australia; ^4^ Department of Nuclear Medicine, The Royal Melbourne Hospital, Parkville, VIC, Australia; ^5^ Faculty of Medicine, Siriraj Hospital, Mahidol University, Bangkok, Thailand; ^6^ PET/CT and Cyclotron Center, Faculty of Medicine, Chiang Mai University, Chiang Mai, Thailand; ^7^ The Florey Institute of Neuroscience and Mental Health, Parkville, VIC, Australia; ^8^ CSIRO Health and Biosecurity, Australian E‐Health Research Centre, Brisbane, QLD, Australia; ^9^ University of Pittsburgh School of Medicine, Pittsburgh, PA, USA

## Abstract

**Background:**

Visual read of Aβ PET remains standard clinical practice. ^18^F‐NAV4694 (NAV) has high affinity for Aβ and low non‐specific binding so may be more accurate than older Aβ tracers. We compared visual read of NAV to ^18^F‐Florbetapir (FBP) PET using standard and AI corrected Centiloid (CL) thresholds as gold standard.

**Method:**

150 participants (71.3±6.0 years) in the AIBL study underwent both scans within 2 years. Scans were read by 6 nuclear medicine physicians blinded to CL. Reader performance was compared against various CL thresholds. CL was measured with CapAIBL using a) the standard method, b) a composite cerebellum plus hemispheric white matter reference region for FBP and c) with an AI correction method for the CL SUVR (DeepSUVR). The DeepSUVR correction was learnt from a separate dataset of longitudinal scans where unexpected temporal changes were penalized and a tracer specific SUVR correction factor was developed for application to single scans.

**Result:**

CL frequency distribution graphs differed for NAV and FBP. Applying the DeepSUVR correction improved the correlation (R^2^) between NAV and FBP CL from 0.82 to 0.94, predominantly by boosting many mid‐range FBP CL values and reducing FBP variance around zero CL (Figure 1). The FBP composite reference region had a similar though less marked effect. The thresholds that produced peak visual read accuracy with standard and DeepSUVR CL methods were 15CL and 11CL for NAV, and 29CL and 21CL for FBP (Figure 2). Mean sensitivity at peak accuracy thresholds was higher in NAV with 97% for standard and 96% for DeepSUVR methods compared to 93% for both methods in FBP. Mean specificity at the same thresholds was similar with 95% for NAV and FBP with standard CL while DeepSUVR produced a specificity of 97% for both tracers.

**Conclusion:**

Visual read of amyloid PET can accurately detect levels of amyloid below the traditional thresholds of 20 or 25CL for a positive scan. Visual read thresholds vary by tracer and reader experience. AI correction of CL with the DeepSUVR method significantly improves the performance of Florbetapir but NAV4694 remains more sensitive to low amyloid levels.